# Work-to-Family Spillover Effects of Workplace Negative Gossip: A Mediated Moderation Model

**DOI:** 10.3389/fpsyg.2020.01612

**Published:** 2020-07-22

**Authors:** Tianyuan Liu, Lin Wu, Yang Yang, Yu Jia

**Affiliations:** ^1^School of Sociology, Wuhan University, Wuhan, China; ^2^School of Journalism and Communication, Wuhan University, Wuhan, China

**Keywords:** workplace negative gossip, psychological distress, work–family conflict, neuroticism, conservation of resource theory, spillover effects

## Abstract

Existing research has found that workplace negative gossip exerts a negative impact on employees and organizations. However, there is a lack of study on the spillover effect of workplace negative gossip on employees’ families. This paper aimed to address this gap in prior literature. Based on resource conservation theory, we chose married employees who perceived or suffered from workplace negative gossip as the subjects and analyzed the effect of workplace negative gossip on their work–family conflict. We adopted a self-reported questionnaire to assess employees’ perception or experience of workplace negative gossip, psychological distress, level of neuroticism, and work–family conflicts. A total of 245 valid employee questionnaires were obtained from two-wave data collection in China. The results of the empirical analysis indicated that workplace negative gossip perceived or suffered by employees has a positive impact on their work–family conflicts, and psychological distress plays a mediating role in the relationship between perceived or suffered workplace negative gossip and employees’ work–family conflict. Furthermore, we found that employees’ level of neuroticism moderates the positive effect of workplace negative gossip and work–family conflict, and it also moderates the mediating effect of workplace negative gossip on employees’ work–family conflict by psychological distress. The conclusion of this paper supported our previous hypotheses. Finally, according to the earlier findings, we discussed the theoretical contributions, practical significance, and limitations of the study and provided some practical suggestions for managers.

## Introduction

Gossip is a key social behavior that nearly everyone working in any organization experiences, hears, and probably contributes to, and it exists in various organizations and places where people live (Dunbar et al., 1997; Foster, 2004; Kniffin and Wilson, 2010; Jiang et al., 2019). The office provides a fertile ground for gossip to spread (Farley et al., 2010). Some scholars pointed out that 14% of the coffee break in the workplace is actually gossip, and approximately 66% of the general communication between employees is talking about other topics of colleagues (Cole and Dalton, 2009). Some earlier studies remarked upon the functions of workplace gossip. Specifically, workplace gossip is a vital channel to facilitate informal communication among employees (Kniffin and Wilson, 2005). Meanwhile, information passed via workplace gossip may explain matters previously unclear to the organization (Noon and Delbridge, 1993; Wu et al., 2012). However, some research has found that workplace gossip is a negative behavior and an extension of abuse, which is often included in a scale that captures broader forms of bullying, such as aggression and harassment (Salin, 2001). Workplace gossip tends to have more adverse effects than positive effects on employees. This is especially true, as competition or “dark behavior” in the workplace has increased (Porath and Pearson, 2010), and most employees have experienced being gossiped about (Snyder et al., 2005). According to a survey of 262 staff in America, nearly 69% of employees admitted to being verbally hurt by their leaders or colleagues in the past year (Fox and Stallworth, 2005).

Workplace negative gossip (WNG) refers to the negative and informal valuation that organizational members discuss or maliciously spread about another member who is absent (Wu L.Z. et al., 2018; Zhou et al., 2019). It mainly includes the following four characteristics, that is, subjective perception, malevolent evaluation, difficult traceability, and rapid dissemination (Ellwardt et al., 2012a; Grosser et al., 2012; Wu L.Z. et al., 2018). Given the previously discussed characteristics of WNG, scholars have urged greater attention to the negative gossip in the workplace (Baumeister et al., 2004; Wu L.Z. et al., 2018). Prior studies focused on the causes of WNG at different dimensions, such as individual level (values, informal status, etc.) and organizational level (integrity of organizational structure, organizational atmosphere, etc.) (Baumeister et al., 2004; Beersma and Van Kleef, 2012; Ellwardt et al., 2012b; Decoster et al., 2013). Some literature has also suggested that WNG can be regarded as an indirect attack or victimization (Beersma and Van Kleef, 2012), which may be harmful to employees’ in-role behavior (Michelson and Mouly, 2000; Kong, 2018; Wu X. et al., 2018; Zhou et al., 2019). WNG damages the reputation of employees and their relationship with colleagues (Shackelford, 1997; Jiang et al., 2019). Exposure to gossip implies that the target does not easily trust others, hampering cooperation with colleagues (Aquino and Thau, 2009). Also, the targets of gossip are likely to suffer greater psychological stress, which exerts an adverse effect on their work motivation, work efficiency, job satisfaction, and innovation (Michelson and Mouly, 2000; Wu L.Z. et al., 2018; Wu X. et al., 2018).

Despite the wealth of existing literature, to our knowledge, little is paid attention to the spillover effect of WNG on employees’ family domain, especially the relationship between WNG and work–family conflict (WFC). Work and family are equally important to employees, especially in Chinese society, where employees’ work and family domains are inseparable and often interact with each other (Liu et al., 2013). Taken together, it is necessary to discuss how and why WNG affects employees’ WFC in the context of Chinese society.

This research bases on the conservation of resource theory (COR) to explain the effect of WNG on WFC in the Chinese context. The COR theory argues that it is vital for individuals to avoid resource loss than to obtain resources (Hobfoll, 1989, 2001). Individuals commonly take multiple roles in their daily life, and resources are always scarce and unevenly distributed. They thus tend to conduct a cognitive assessment of the return of each role and the situation of resource loss in advance to determine which roles to give up or invest in. According to the previously mentioned principles, if employees decide to consume numerous resources to cope with WNG in work life, maybe the remaining resources they could devote to family life are scarce, which positively impacts their WFC.

Meanwhile, WNG represents a resource loss (Wu X. et al., 2018). The COR theory posits that stress and distress occur when resources are lost. When the loss of the resource is greater or exhausted, individuals will enter a defensive mode to conserve resources, appearing aggressive and in an irrational behavior (Hobfoll et al., 2018). By drawing on the COR theory, we thus suggest that psychological distress plays a mediating role in the relationship between WNG and WFC.

Also, to our knowledge, little do we know about whether personality traits provide a boundary condition of gossip’s effects. This study aims to explore the “dark side” in the workplace. Neuroticism, a kind of the big five personality traits, is related to negative feelings such as anxiety, frustration, and emotional instability. We thus use neuroticism as a moderator. More precisely, we propose that high neuroticism may strengthen the relationship between WNG and psychological distress and the mediating effect of psychological distress on the relationship between WNG and WFC.

**FIGURE 1:**
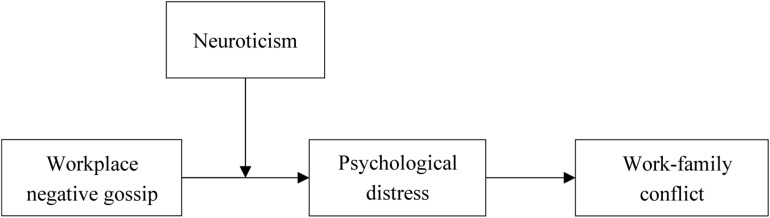
The conceptual mode of this research.

Taken together, this study makes three contributions to the WNG and family life literature. First, existing research has little attention to the effect of WNG on family life. To fill the gap in existing literature, we apply the COR theory to argue why WNG from the perspective of resource loss could cause WFC, especially in the Chinese cultural context. Second, we examine how WNG conduces to WFC. Specifically, psychological distress plays a mediating role between WNG and WFC. Finally, by testing the moderating mechanism of neuroticism in the earlier mentioned relationships, we reveal that the boundary condition of the effect of WNG on WFC. Figure 1 is the conceptual model.

## Theory and Hypothesis

### Main Effect of Workplace Negative Gossip on Employees’ Work–Family Conflict

Work and family are two crucial components of employees’ life (Kossek and Ozeki, 1998; Anderson et al., 2002). WFC represents a particular type of inter-role conflict in which the role pressures from the work and family domains are incompatible in some aspects (Greenhaus and Beutell, 1985). A review of related literature suggests that WFC is generally divided into three major forms, including time-based conflict, strain-based conflict, and behavior-based conflict (Greenhaus and Beutell, 1985; Liu et al., 2013). Time-based conflict takes place when individuals spend time on the work role and make it difficult to perform their family duties. Strain-based conflict is produced when some strain symptoms (i.e., stress, tension, anxiety, etc.) caused by the work role affect one’s performance in family role. Behavior-based conflict exists when a person is unable to adjust behavior from work to meet with the expectations regarding behavior in the family role.

Workplace negative gossip is a dilemma faced by employees in their office life. As they make use of limited resources to cope with gossip about themselves to change the situation, it means their resources are consumed (Liu et al., 2020). In other words, WNG represents a kind of resource loss (Wu X. et al., 2018). The COR theory argues that protection of remaining resources is a priority for those individuals who suffer a resource loss (Hobfoll, 1989, 2001). Along this line, given the issue of limited resources, if employees have devoted more time and energy to deal with negative gossip in the work domain, they would tend to protect their remaining resources in the family domain. An effective way in which employees seek to protect personal resources is to reduce the amount of energy invested in their families. Meanwhile, the COR theory suggests that loss will have a significantly greater impact, and the lack of personal resources will bring about difficulties handling their daily life (Hobfoll, 1989, 2001). Therefore, we could propose that employees’ behaviors of priority protection of remaining resources and the loss of their resources will make it difficult to balance work and family roles effectively, resulting in WFCs.

More precisely, employees suffering from WNG may adopt some approaches such as hard work or attendance at group activities to change the gossipers’ negative assessment of themselves. Thus, they may face working hours overload, and WFC is related to work time per week (Schafer, 1980). Given the priority of resources protection, they are more likely to reduce the investment in time or energy for family role, resulting in time-based WFC. Moreover, the targets of gossip have already done much emotional labor for coping with gossip in the workplace. Therefore, following the principle of protection priority, employees tend to have a cold and detached attitude toward their partners or relatives to preserve individual psychological resources when they return home, thereby generating strain-based conflict. Furthermore, employees who have been exposed to WNG are likely to display warmth and emotionality to repair their reputation in their interactions with those gossipers. Consequently, for the conservation of personal resources, they cannot meet family expectations that a person to be warm and nurturant, leading to behavior-based conflict. Thus, we propose the following hypothesis:

Hypothesis 1 (H1): *WNG will yield WFC of employees.*

### Mediating Effect of Psychological Distress

Psychological distress, an unpleasant state, is often associated with negative thoughts and emotions, such as anxiety, fear, depression, etc. (Barnett and Brennan, 1997). Psychological distress takes place when individuals experience the stress and strain of work and family life and emotional trauma (Keashly and Harvey, 2005).

The COR theory provides a theoretical explanation for whether and in what situations WNG leads to psychological distress. The COR theory posits that individuals prefer to retain their existing resources. However, when personal resources are threatened, lost, and believed to be unstable, people are likely to suffer psychological distress (Hobfoll, 1989, 2001; Hobfoll and Lilly, 1993). Prior researches have exhibited evidence for the interpretation that stressors are regarded as a threat to individual resources, which finally produces greater psychological distress (Almeida, 2005).

Generally, China is seen as a collectivist society. Most people thirst for properly dealing with personal relationships and expect to be acknowledged and treated kindly (Hofstede, 1991). In this cultural context, when employees suffer from WNG, they will immediately attempt to deploy resources to improve their situation (Wu X. et al., 2018). We thus suggest that WNG represents resource loss and interpersonal stressors (Shackelford, 1997; Liu et al., 2020). This is mainly based on the following two considerations. Firstly, exposure to negative gossip implies that the targets tend to be angry and frustrated, especially if the gossip is fake and hostile. They could consume substantial personal resources to address the situation (Wu X. et al., 2018; Liu et al., 2020), such as identifying the gossiper, justifying their behaviors, and elucidating the truth, etc. Secondly, employees whose reputation has been damaged by gossip are less likely to obtain resources from superiors or colleagues to make up their resource loss, even increasing the possibility of resource depletion (Wu et al., 2012). For example, when the targets would like to approach colleagues and increase social connections actively, colleagues generally have an evasive or indifferent attitude. This not only results in no return on the resources invested by individuals but also speeds up the loss of their psychological resources such as lowering self-esteem, increasing anxiety, etc. Taken together, according to the COR theory, WNG, as a resource loss, is strongly related to psychological distress (Chandra and Robinson, 2009).

The COR theory also posits some important corollaries. Especially corollary 2 provides a theoretical explanation for scholars to study how WNG yields WFC. Corollary 2 is called “loss spirals” because resource loss is more powerful than resource gain and because stress and distress occur when resources are lost. At each iteration of the stress spiral, the individual’s resources used to offset resource losses gradually decrease, but the momentum and magnitude of resource losses are still increasing, leading to further damage to future resources (Hobfoll et al., 2018). When the resource loss is greater or exhausted, individuals will enter a defensive mode to conserve resources, appearing aggressive and in an irrational behavior (Hobfoll et al., 2018). Exposure to WNG implies that employees have already faced resource loss and suffered a great deal of psychological distress. Under the influence of the resource loss spiral, psychological stress and distress further increased their resource loss, making them face the threat of resource exhaustion. Consequently, they often choose a defensive strategy to conserve their resource reserves. Employees’ work and family domains are closely linked, especially in China. They tend to conserve their resources by adopting the defensive posture in their family life. For example, they tend to be passive avoidant to avoid the emotional labor in family life. This may conduce to tensions, disputes, and disharmony in family relations, which in turn cause WFC.

Overall, given the positive effect of WNG on the psychological distress and the positive role of the psychological distress in aggravating WFC, we propose the following hypothesis:

Hypothesis 2 (H2): *Psychological distress mediates the positive relationship between WNG and WFC.*

### Moderating Effect of Neuroticism

In this study, we mainly aim to explore the adverse effects of the “dark side” in the workplace. As a result, we regard neuroticism, a kind of the big five personality traits, as a moderator rather than other dimensions of the big five personality traits. This is because only neuroticism is directly relevant to unfavorable feelings, such as anxiety, depression, and self-doubt, whereas other traits are related to positive emotions or behaviors. Consequently, we adopt the COR theory to analyze the conditions under which neuroticism is likely to strengthen or weaken the influence of WNG.

The COR theory argues that resources comprise those objects such as individual characteristics, conditions, or energies that are valued in their own right or that are valued because they play vital roles in obtaining or production of valued resources (Diener and Fujita, 1995; Hobfoll, 2001). Resources can be specifically divided into four categories: material resources, conditional resources, personality traits, and energy resources (Hobfoll et al., 2018). These resources can not only meet individual needs but also help them accurately identify and socially locate themselves. In line with this definition and classification of resource, we could suggest that neuroticism represents a characteristic resource that individuals possess.

Of the primary dimensions of personality, neuroticism is typically defined as a tendency toward anxiety, depression, self-doubt, and other negative feelings. It is closely linked with one’s emotional stability. Lower levels of neuroticism indicate that the individual possesses a strong power of emotional control and regulation. In other words, individuals with low levels of neuroticism have abundant psychological resources, such as emotionally stable. Conversely, higher levels of neuroticism represent those individuals who usually suffered mood swings due to the effect of external factors. That is, individuals with high levels of neuroticism are lack of psychological resources.

The COR theory posits that individuals with greater resources are less vulnerable to resource loss and more capable of resource gain. On the contrary, those who lack resources are more susceptible to resource loss and less capable of resource gain, and their willingness to protect remaining resources is stronger than their awareness of acquiring surplus resources (Hobfoll et al., 2018). Following this theoretical logic, we suggest that when employees with different levels of neuroticism experience resource loss, they are more likely to show diverse perceptions of this adverse event, thus adopting differentiation strategy. More specifically, employees with high neuroticism act more sensitive to resource loss (i.e., WNG) for lack of psychological resources, and they are likely to constantly reinforce resource loss and magnify negative perceptions of themselves (Li et al., 2016; Lu et al., 2017; Decuypere et al., 2018). Consequently, they tend to be in the grip of psychological distress, such as anxiety, tension, and emotional instability. Instead, individuals with low neuroticism have considerably psychological resources. They thus are insensitive to the adverse effects of recourse loss. They could exert their ability and resource to maintain emotional stability and show positive aspects in their work attitudes and behaviors, reducing the psychological distress caused by resource loss, that is, exposure to WNG. Thus, the following hypothesis is proposed:

Hypothesis 3 (H3): *Neuroticism moderates the relationship between WNG and psychological distress, such that the positive relationship is stronger when the employee possesses higher neuroticism.*

Also, based on the previously discussed assumption that the positive impact of WNG on psychological distress is stronger when the employee has higher neuroticism and that psychological distress is positively related to WFC, it is logical to speculate that the positive indirect effect of WNG on WFC via psychological distress will be stronger when an employee has higher neuroticism. Hence, we propose that:

Hypothesis 4 (H4): *Neuroticism moderates the indirect effect of WNG on WFC via psychological distress. Specifically, the indirect effect will be strengthened when the employee possesses higher neuroticism.*

## Materials and Methods

### Sample and Procedures

The data used in this paper were collected with the help of a professional research company, which built a large database containing information from numerous enterprises and employees in China. The data company randomly selected 1,000 employees in its database as the initial subjects. Data were collected at different time points for reducing the possibility of common method bias. Existing literature on resource loss has used different time lags, including 6 weeks (Ritter et al., 2016), 3 months (Uglanova and Staudinger, 2013), 6 months (Matthews et al., 2014), and even a few years (Knecht et al., 2011). There is no one right temporal lag that can be universally recommended (Menard, 2002). Some scholars indicated that shorter intervals in studies should be considered because longer lags could miss the manifestation window and impact the true relationship (Tetrick and Buffardi, 2006). Consequently, we use 3-month gaps in this study. That is, we sent the questionnaires to 1,000 participants at two different times over a span of 3 months. Meanwhile, we coded the questionnaires before distributing the survey to match the responses to different times.

In the first-wave survey, we distributed the questionnaire to the respondents by email, which mainly included the basic information of the subjects. We also surveyed participants if they had perceived or experienced WNG during the past 6 months and their marital status. Given the core goal of this study, we only retained questionnaires where items of the experience of WNG and marital status were both answered “yes.” In other words, married employees who suffered from WNG were the ones we ultimately studied. Specifically, in the first-wave survey, we surveyed a total of 1,000 employees and received 821 questionnaires, including 660 married samples. Of the 660 married samples, 502 samples perceived or experienced WNG. After removing missing values of some variables, we obtained 445 valid questionnaires in the first wave. The second-wave data collection was conducted 3 months later; 455 employees who had completed first-wave questionnaires were surveyed again, investigating their personality traits, psychological distress, and WFC. We received 268 responses, for a response rate of 58.9%. After sorting out the questionnaires, we finally obtained 245 valid results, which formed the basis of data analysis in this paper.

Of the 245 usable samples, there were 105 female and 140 male participants. About 26.53% had state-owned jobs, and the remaining employees had non-state-owned jobs. Only 32.24% were in management positions. The average age of all participants was 32.67 years old (*SD* = 7.26). Of the employees, 66.9% lived with their parents, and the rest did not. With regards to education level, the proportions of those with high school degrees or below, those with associate degrees, and those with master’s degrees or above were less than 3, 10.61, and 8.98%, respectively. The number of employees with bachelor’s degrees was the largest, accounting for 77.96%. Also, the average employee changed jobs about 3.39 times (*SD* = 1.50).

### Measure

#### Workplace Negative Gossip

We measured the employees’ perceptions of WNG by the three-item scale from the work of Wu L.Z. et al. (2018). Example items were “During the past 6 months, others have spread damaging information about you in office,” “During the past 6 months, others have communicated unfavorable gossip about you in office,” and “During the past 6 months, others have made negative allegations about you in office.” All adopted a seven-point Likert scale, ranging from 1 (never) to 7 (daily). The scale’s reliability was 0.900.

#### Psychological Distress

We adopted a six-item scale developed by Kessler et al. (2002) to measure psychological distress. Respondents were asked to answer how often they generally experienced some special emotions or feelings during the last 30 days, including nervousness, hopelessness, restlessness or fidgetiness, depression (nothing could cheer you up), struggling (everything was an effort), and worthlessness. Each item was measured on a seven-point scale (1 = never, 7 = daily). Cronbach’s alpha for this scale was 0.902.

#### Neuroticism

Shafer (1999) designed a brief bipolar rating scale to measure the five-factor model of personality, including extraversion, neuroticism, conscientiousness, agreeableness, and openness. We applied one subscale as an indicator of neuroticism, which consisted of six items such as “at ease-nervous,” “calm-anxious,” “unworried-fearful,” etc. Respondents were asked to indicate the extent of agreement, which ranged from (1) strongly agree to (7) strongly disagree. The scale’s reliability was 0.939.

#### Work–Family Conflict

Work–family conflict was assessed with five items applied by Anderson et al. (2002). Respondents were asked to indicate the extent to which one’s work interfered with one’s family. Sample items included: “Have you not had enough time for yourself because of your job?,” “Have you not been in as good of a mood as you would like to be at home because of your job?,” and so on. We used a seven-point Likert scale (1 = very often, 7 = never). To maintain consistency of data direction, we reverse-coded those items, that is, higher numbers denote more frequent experiences of one’s work interfering with one’s family. Cronbach’s alpha for this scale was 0.884.

#### Control Variables

To eliminate potential confounding effects, we took demographic information of the employees, including age, gender, education level, and living with parents (Byron, 2005), as control variables. Meanwhile, following existing research on WNG, we also controlled for employees’ position, number of job changes, and ownership.

## Results

### Confirmatory Factor Analysis

Before testing the previously discussed hypotheses, we needed to conduct confirmatory factor analyses with Mplus7 to verify the discriminant validity because all our core variables were from the same questionnaire. The indicators recognized by most scholars were adopted to judge the model fit, including χ^2^/df, comparative fit index (CFI), Tucker–Lewis index (TLI), and root mean square error of approximation (RMSEA). When the values of CFI and TLI are greater than 0.9, the value of χ^2^/df is between 1 and 5, the value of RMSEA is less than 0.08, and the overall fitness of the model is better and can be accepted. We planned to pick the optimal fit model by comparing the assumed factor models.

We first tested the single-factor model that contained all of variables, which yielded a poor fit (χ^2^ = 1,956.067, χ^2^/df = 11.506, CFI = 0.488, TLI = 0.427, and RMSEA = 0.207). Then, we examined other alternative factor models, two two-factor models, and five three-factor models, which did not provide good fits either. As we assumed, the four-factor model contributed the best fit to the data: χ^2^ = 230.789, χ^2^/df = 1.407, CFI = 0.981, TLI = 0.978, and RMSEA = 0.041 (Table 1), demonstrating that the discriminant validity of this study variables was high and effective.

**TABLE 1:** Results of the confirmatory factor analysis for the main variables.

Factor models	χ^2^	df	χ^2^/df	CFI	TLI	RMSEA
Single-factor mode l	1,956.067	170	11.506	0.488	0.427	0.207
Two-factor model 1	1,459.128	169	8.634	0.630	0.584	0.177
Two-factor model 2	1,267.798	169	7.502	0.685	0.646	0.163
Three-factor model 1	1,512.979	167	9.060	0.614	0.561	0.181
Three-factor model 2	888.937	167	5.323	0.793	0.764	0.133
Three-factor model 3	846.932	167	5.071	0.805	0.778	0.129
Three-factor model 4	802.730	167	4.807	0.818	0.792	0.125
Three-factor model 5	660.284	167	3.954	0.858	0.839	0.110
Four-factor model	230.789	164	1.407	0.981	0.978	0.041

*Single-factor model: WNG + NE + PD + WFC; two-factor model 1: WNG + WFC and NE + PD; two-factor model 2: WNG + PD + WFC and NE; three-factor model 1: WNG, WFC + NE, and PD; three-factor model 2: WNG + WFC, NE, and PD; three-factor model 3: WNG, WFC + PD, and NE; three-factor model 4: WNG, NE + PD, and WFC; three-factor model 5: WNG + PD, NE, and WFC; four-factor: WNG, NE, PD, and WFC. *WNG, workplace negative gossip; NE, neuroticism; PD, psychological distress; WFC, work–family conflict*.*

### Descriptive Statistics

Table 2 shows the result of descriptive statistics and correlations among all variables, including the means, standard deviations, and the correlation coefficients. Obviously, there were close relationships between the core variables in the study. WNG was positively correlated with psychological distress (*r* = 0.393, *p* < 0.01) but negatively related to WFC (*r* = −0.274, *p* < 0.01). As we expected, psychological distress was significantly associated with neuroticism (*r* = 0.575, *p* < 0.05) and WFC (*r* = 0.356, *p* < 0.01). Also, neuroticism and WFC were positively related to one another (*r* = 0.153, *p* < 0.05).

**TABLE 2:** Descriptive statistics and correlations among all variables.

Variables	1	2	3	4	5	6	7	8	9	10	11	12	13	14
(1) Workplace negative gossip	1													
(2) Psychological distress	0.393**	1												
(3) Neuroticism	–0.034	0.575*	1											
(4) Work–family conflict	−0.274**	0.356**	0.153*	1										
(5) Age	–0.042	0.001	0.037	–0.060	1									
(6) Gender	0.219**	0.417**	0.187**	0.208**	–0.085	1								
(7) Education 1	0.263*	0.229**	0.199**	0.096	0.040	–0.023	1							
(8) Education 2	0.024	0.069	0.010	0.085	0.100	0.031	–0.055	1						
(9) Education 3	−0.144*	–0.078	–0.042	–0.109	–0.073	0.017	−0.298**	−0.648**	1					
(10) Education 4	0.041	–0.085	–0.057	0.015	–0.023	–0.045	–0.050	–0.108	−0.591**	1				
(11) Living with parents	0.185**	0.566**	0.252**	0.205**	0.111	0.263**	0.109	0.044	–0.079	0.008	1			
(12) Position	–0.017	–0.022	0.035	0.016	−0.142*	0.068	–0.053	0.216**	–0.076	–0.095	–0.107	1		
(13) The number of job-hopping	–0.008	−0.134*	−0.148*	–0.059	−0.217**	–0.123	0.012	0.141*	−0.132*	0.033	–0.066	0.031	1	
(14) Ownership	–0.057	0.057	0.087	0.050	0.096	0.016	0.024	–0.027	–0.015	0.038	0.048	0.060	−0.341**	1
Mean	3.820	3.699	3.214	4.094	32.673	0.571	0.024	0.106	0.780	0.090	0.669	0.322	3.388	0.265
*SD*	0.913	1.189	1.272	1.059	7.262	0.496	0.155	0.309	0.415	0.286	0.480	0.468	1.499	0.442

N = 245. * P < 0.05, ** P < 0.01. Sex: 1—male, 0—female; Ling with parents: 1—yes, 0—no; Ownership: 1—state-owned, 0—non-state-owned; Position: 1—manager, 0—employee; Education 1: high school degrees or below, Education 2: associate degree, Education 3: bachelor’s degree, Education 4: master’s degree or above.

### Hypothesis Testing

We used hierarchical regression analyses to test Hypotheses 1 and 2. Hypothesis 1 of this study predicts that WNG yields employees’ WFC. As shown in Table 3, WNG was positively associated with employees’ WFC (β = 0.245, *p* < 0.01; Model 4), supporting Hypothesis 1. Also, Hypothesis 2 of this study posits that psychological distress mediates the positive relationship between WNG and WFC. First, the control variables and the independent variable were entered into Model 1. Regression result of Model 1 indicated that WNG had a positive relationship to psychological distress (β = 0.294, *p* < 0.01; Model 1). Second, Model 5 is that we added the mediating variable based on Model 1. Its results represented that psychological distress was positively correlated with WFC, as WNG is controlled (β = 0.225, *p* < 0.01; Model 5). Meanwhile, when psychological distress is controlled, WNG is positively related to WFC (β = 0.179, *p* < 0.05; Model 5). In conclusion, according to the causal steps approach used by Baron and Kenny (1986), we propose that psychological distress plays a mediating role in the relationship between WNG and WFC, supporting Hypothesis 2.

**TABLE 3:** Results of hierarchical regression analyses.

Variables	Psychological distress			Work–family conflict				
		
	Model 1	Model 2	Model 3	Model 4	Model 5	Model 6	Model 7	Model 8
**Control variables**								
Education 2	−0.775*	–0.016	0.592	0.061	0.235	0.220	1.038**	0.946**
Education 3	−0.985***	–0.197	0.406	–0.254	–0.032	–0.088	0.723	0.660
Education 4	−1.294***	–0.461	0.195	–0.179	0.113	–0.004	0.879*	0.849
Ownership	0.059	0.037	–0.015	0.108	0.095	0.103	0.034	0.367
Hop	–0.068	–0.025	–0.021	–0.036	–0.021	–0.027	–0.023	–0.019
Position	–0.013	–0.073	–0.071	–0.005	–0.002	–0.018	–0.016	–0.005
Living with parents	1.103***	0.861***	0.839***	0.289**	0.041	0.238	0.209	0.079
Age	–0.009	–0.008	–0.007	–0.012	–0.010	–0.012	–0.011	–0.010
Sex	0.560***	0.396***	0.375***	0.240*	0.114	0.206	0.178	0.120
**Independent variable**								
Workplace negative gossip	0.294***	0.391***	–0.094	0.245***	0.179**	0.266***	−0.387**	−0.372**
**Moderator**								
Neuroticism		0.425***	–0.035			0.089	−0.529***	−0.524***
**Interaction 1**								
Workplace negative gossip* Neuroticism			0.131***				0.176***	0.156***
**Mediator**								
Psychological distress					0.225***			0.156*
_cons	2.993***	0.592	1.733***	2.847***	2.847***	3.016***	4.552***	4.282***
*r*^2^	0.490	0.665	0.687	0.163	0.163	0.140	0.190	0.199

N = 245. * p < 0.1, ** p < 0.05, *** p < 0.01.

Moreover, we adopted the bootstrapping procedure with 5,000 subsamples to further examine the main and mediating effects. If the 95% confidence interval (CI) does not contain zero, this means the effects are significant. Table 4 shows the analysis results. In particular, WNG was positively associated with WFC (β = 0.364, *SE* = 0.113, 95%, CI = [0.181, 0.555], CI did not include zero), and Hypothesis 1 was tested again. The results also revealed a significant indirect effect of WNG on WFC, and the 95% CI ranged from 0.076 to 0.280 (CI did not include zero). Hence, these findings provided initial support for Hypothesis 2.

**TABLE 4:** Non-standardized mediation analysis results.

Model paths	Estimate	*SE*	BC 95% CI	
			
			Lower	Upper
**Total effect**				
WNG → WFC	0.364	0.113	0.181	0.555
**Direct effect**				
WNG → PD	0.547	0.093	0.388	0.692
PD → WFC	0.296	0.098	0.139	0.460
WNG → WFC	0.202	0.120	0.001	0.392
**Indirect effect**				
WNG → PD → WFC	0.162	0.061	0.076	0.280

*BC, biased corrected (5,000 bootstrapping sample). Control variables (age, sex, education 2, education 3, education 4, living with parents, position, the number of job-hopping, and ownership) were added to the non-standardized mediation analysis. WNG, workplace negative gossip; PD, psychological distress; WFC, work–family conflict*.

**FIGURE 2:**
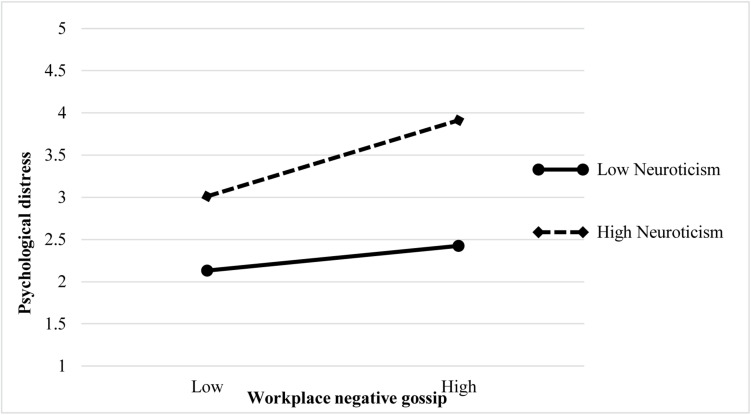
Interactive effect of neuroticism on the relationship between workplace negative gossip and employees’ psychological distress.

We applied the moderated causal step approach of analysis to examine Hypothesis 3, which expects that neuroticism plays a moderating role in the relationship between WNG and psychological distress. As Table 3 indicated, interaction one term was significantly and positively related to psychological distress (β = 0.131, *p* < 0.01, Model 3), supporting Hypothesis 3. Following the procedure recommended by Stone and Hollenbeck (1989), we further computed the slopes using one SD above (high level of neuroticism) and below (low level of neuroticism) the mean of the moderating variable and then plotted the moderation patterns. As shown in Figure 2, we found the moderation patterns to be consistent with Hypothesis 3. When employees possessed a high level of neuroticism, WNG exerted a stronger positive influence on psychological distress than the individuals enjoyed a low level of neuroticism. Therefore, Hypothesis 3 was verified again.

We utilized PROCESS macro for SPSS 23.0 with 5,000 bootstrap samples to test Hypothesis 4. The results reported in Table 5 suggested an indirect effect of WNG on employees’ WFC via psychological distress whether the level of neuroticism was high or low and that the indirect effect was significant and positive. Specifically, when employees possessed high neuroticism, the indirect effect was enhanced (low = 0.073, mean = 0.102, and high = 0.160, CI did not include zero). By combining these analyses, Hypothesis 4 was well demonstrated.

**TABLE 5:** Moderated mediation results.

Moderator variable	Estimate	*SE*	BC 95% CI	
			
			Lower	Upper
Emotional intelligence low	0.073	0.035	0.007	0.144
Emotional intelligence mean	0.102	0.031	0.045	0.167
Emotional intelligence high	0.160	0.053	0.072	0.040
Index	0.035	0.023	0.006	0.099

*BC, biased corrected (5,000 bootstrapping sample). Control variables (age, sex, education 2, education 3, education 4, living with parents, position, the number of job-hopping, and ownership) were added to the non-standardized mediation analysis*.

## Discussion

This study mainly explores the impact of WNG perceived and experienced by employees on their WFCs as well as the specific influencing mechanism. Two hundred forty-five employees from Chinese companies were investigated for data analysis, and results show that WNG perceived or suffered by employees has a positive impact on employees’ WFCs through the mediating variable of psychological distress. Meanwhile, employees with high levels of neuroticism have a more adverse effect on their psychological distress by the WNG that they perceived or experienced compared with those with low levels of neuroticism. Moreover, neuroticism moderates the indirect effects of WNG on the WFCs of employees through psychological distress. Also, our research has some theoretical and practical significance, which we will discuss in the next section.

### Theoretical Implications

In the current increasingly competitive workplace, WNG is a common phenomenon. Scholars have carried out plenty of research on the possible impact of WNG on employees and organizations, forming a rich body of literature. However, there is a lack of research on the possible impact of WNG on the employees’ families as well as the mediating mechanism. To fill in these gaps, we regarded it as the main research issue and conducted an empirical analysis in the Chinese context. Therefore, the results of this study have theoretical significance. First, according to resource conservation theory, this study discusses the spillover effect of WNG on the family life of employees, that is, the impact on the conflict of working families. Although there is a large number of studies on the adverse effects of negative gossip in the workplace, they mainly focus on the impact of WNG on job motivation, job performance, job satisfaction, etc. (Kong, 2018; Babalola et al., 2019). The results of this study extend the impact of WNG from the individual level of the employee to the family level, thereby opening up a black box between WNG and WFCs.

Second, based on the perspective of work stress, this study reveals an important mediating mechanism of how WNG leads to WFCs of employees. In other words, WNG leads to WFC through the mediating role of psychological distress. At the same time, perceived or suffered WNG exacerbates the psychological distress of employees, which means that employees’ psychological distress may not only come from structural factors, such as organizational design, job content, etc. but may also be influenced by interpersonal relationship among employees within the organization. Thus, our study also helps advance the employees’ psychological distress literature.

Finally, the results of this study show that neuroticism, a big five personality trait, moderates the relationship between WNG and WFC. Specifically, employees with lower levels of neuroticism have a stronger ability to control and regulate emotions and possess less extreme emotions. Therefore, they can properly deal with WNG, slow down their emotional distress, and reduce the possibility of causing WFCs. On the contrary, employees with higher levels of neuroticism have a lack of stability in their emotions and a lower ability to adjust their own emotions, and they are prone to negative psychological problems. Once they encounter WNG, they will have great psychological distress, exacerbating WFCs. In summary, the research results confirm a boundary condition that WNG causes psychological distress, further expanding the research on WNG.

### Practical Implications

In practical terms, it is necessary for organizations and managers to recognize that WNG may lead to WFCs of employees, which in turn will affect the behavioral expression and work efficiency of employees in the workplace, even negatively affecting business performance. Therefore, this study proposes three specific methods to help organizations and managers pay attention to avoiding WNG, reducing WFCs of employees.

The first method is taking positive measures to tighten or close the transmission channels of WNG. Existing literature indicates that an important cause of the occurrence of gossip is when individuals discover the uncertainty of the environment in which they are located (Rosnow et al., 1988; DiFonzo and Bordia, 2007). Considering the importance of environmental uncertainty, organizations or managers should make changes accordingly, such as keeping transmission channels for information unimpeded within organizations, revealing important information about employees’ interests promptly, preventing ambiguous environments from occurring, and reducing opportunities for rumors to spread within the organization. At the same time, organizations should establish a cultural atmosphere of zero tolerance toward WNG. Organizations must apply certain disciplinary measures to employees who create and disseminate negative gossip and cause significant adverse effects within the organization, such as destroying the trust of colleagues and jeopardizing internal unity. Moreover, organizations should encourage social interaction between employees within the organization, build friendliness, intimacy, and cooperation among colleagues, and avoid undesirable workplace behavior. Also, organizational rules and norms can apply pressure against the spread of WNG (Michelson et al., 2010), and organizations or managers can issue formal organizational rules and norms to constrain and manage employees’ behavior within the organization.

The second path is to help employees improve their psychological endurance capacities and resistance abilities as much as possible. A large number of studies have indicated that the psychological endurance capacities of employees are closely related to their job performance. In the face of adverse external shocks, individuals with a higher psychological endurance capacity can maintain positive and stable emotions, avoiding external shocks that have adverse effects on their work and family; on the contrary, individuals with a lower psychological endurance capacity may fall into nervousness, fidgetiness, or depression and cannot alleviate the impact of adverse external shocks on their own family and work. Therefore, organizations can provide non-profit consulting services for employees with low psychological endurance capacities, help them to properly face external shocks, properly resolve the possible damage caused by shocks, and help them improve themselves and reduce the hazard to their work and organization. At the same time, all employees should receive regular psychological training to help themselves know how to cope with stress and respond to WNG.

Thirdly, neuroticism level is one of the main personality traits of individual employees, and it is also an important catalyst for the adverse effects of WNG. People with high levels of neuroticism are very sensitive to WNG. This has practical revelations for managers and organizations in that when conditions permit, managers and organizations can measure and analyze personality traits in the recruitment process and can regard their personality traits as an important factor in recruitment. For employees with high levels of neuroticism who have already been recruited, enterprises can help employees gradually improve their psychological status, maintain healthy and stable emotions, and cultivate a good work and life mentality through the organization of public welfare psychological activities.

### Limitations and Future Research

Although our research has some theoretical and practical implications, there are some limitations. First of all, we take corporate employees as the object of study and do not make a sex distinction. As is well known, maintaining the balance between work and family is a difficult problem for women in the workplace. WFCs are common among women in the workplace, and the only difference is the size of the conflict. Women workers have long been in WFCs. Have they formed some special responses, and can these measures help them cope with WNG? Hence, future research can focus on the working female group and conduct a comparative study with the working male group. Secondly, this study mainly considers the negative gossip perceived or experienced by employees at work from their colleagues. According to existing literature, gossip may also come from those in power (Gkorezis et al., 2014; Kuo et al., 2018). Employees may suffer from negative gossip from their supervisors, which may have a greater impact on individual employees and their families. After all, leaders have higher management power than the colleague. Therefore, further research can explore the impact of WNG from different sources on employees’ WFCs. Finally, our research is carried out under the Chinese context, and the sample size is not enough. The Chinese situation is special in that employees’ work-life and family-life are closely related and have a strong overflow effect. However, in developed countries where a more detailed labor welfare system has been established, there is a clear boundary between work and family. Do employees’ perceived or suffered WNG cause conflicts between their work and families? This issue requires further study in the future. Also, future research wants to delve deeper into the results by using a bigger sample size.

## Conclusion

Previous research generally pays attention to the adverse impact of WNG on employees’ job performance and organizational efficiency, but few studies have explored the impact of WNG on WFCs. This article explores the relationship between the two in the Chinese situation. The empirical analysis validates our hypothesis that psychological distress plays a mediate role between WNG and employees’ WFCs and that the neuroticism of employees is an important moderating factor. Also, the findings of our research provide a perspective for understanding the harms of WNG and can help organizations recognize the role of WNG and reduce the adverse effects through relevant initiatives.

## Data Availability Statement

The datasets for this article are not publicly available due to restrictions set by the data holder. Requests to access the datasets should be directed to ltyuan@whu.edu.cn.

## Ethics Statement

The studies involving human participants were reviewed and approved by Ethics Committee, School of Sociology, Wuhan University. The patients/participants provided their written informed consent to participate in this study.

## Author Contributions

TL contributed in writing the original draft, conceptualization, data curation, formal analysis, and methodology. LW contributed in resources, data collection, and supervision of the manuscript. YJ and YY contributed in data curation, methodology, review, and editing. All authors contributed to the article and approved the submitted version.

## Conflict of Interest

The authors declare that the research was conducted in the absence of any commercial or financial relationships that could be construed as a potential conflict of interest.

## References

[B1] Almeida D. M. (2005). Resilience and vulnerability to daily stressors assessed via diary methods. *Curr. Direct. Psychol. Sci.* 14 64–68. 10.1111/j.0963-7214.2005.00336.x

[B2] Anderson S. E. Coffey B. S. Byerly R. T. (2002). Formal organizational initiatives and informal workplace practices: links to work–family conflict and job-related outcomes. *J. Manag.* 28 787–810. 10.1016/s0149-2063(02)00190-3

[B3] Aquino K. Thau S. (2009). Workplace victimization: aggression from the target’s perspective. *Ann. Rev. Psychol.* 60 717–741. 10.1146/annurev.psych.60.110707.163703 19035831

[B4] Babalola M. T. Ren S. Kobinah T. Qu Y. E. Garba O. A. Guo L. (2019). Negative workplace gossip: its impact on customer service performance and moderating roles of trait mindfulness and forgiveness. *Int. J. Hospital. Manag.* 80 136–143. 10.1016/j.ijhm.2019.02.007

[B5] Barnett R. C. Brennan R. T. (1997). Change in job conditions, change in psychological distress, and gender: a longitudinal study of dual-earner couples. *J. Organ. Behav.* 18 253–274. 10.1002/(sici)1099-1379(199705)18:3<253::aid-job800<3.0.co;2-7

[B6] Baron R. M. Kenny D. A. (1986). The moderator–mediator variable distinction in social psychological research: conceptual, strategic, and statistical considerations. *J. Pers. Soc. Psychol.* 51 1173–1182. 10.1037/0022-3514.51.6.1173 3806354

[B7] Baumeister R. F. Zhang L. Q. Vohs K. D. (2004). Gossip as cultural learning. *Rev. Gen. Psychol.* 8 111–121. 10.1037/1089-2680.8.2.111

[B8] Beersma B. Van Kleef G. A. (2012). Why people gossip: an empirical analysis of social motives, antecedents, and consequences. *J. Appl. Soc. Psychol.* 42 2640–2670. 10.1111/j.1559-1816.2012.00956.x

[B9] Byron K. (2005). A meta-analytic review of work-family conflict and its antecedents. *J. Vocat. Behav.* 67 169–198. 10.1016/j.jvb.2004.08.009

[B10] Chandra G. Robinson S. (2009). “They’re talking about me again: the impact of being the target of gossip on emotional distress and withdrawal,” in *Proceedings of the Academy of Management Conference*, Chicago.

[B11] Cole J. Dalton J. (2009). “Idle women’s chat? Gender and gossip. Social Section,” in *Proceedings of the Annual Conference of the British Psychological Society*, (Kent, UK: University of Kent).

[B12] Decoster S. Camps J. Stouten J. Vandevyvere L. Tripp T. M. (2013). Standing by Your Organization: the Impact of Organizational Identification and Abusive Supervision on Followers’. Perceived Cohesion and Tendency to Gossip. *J. Bus. Ethics* 118 623–634. 10.1007/s10551-012-1612-z

[B13] Decuypere A. Audenaert M. Decramer A. (2018). When mindfulness interacts with neuroticism to enhance transformational leadership: the role of psychological need satisfaction. *Front. Psychol.* 9:2588. 10.3389/fpsyg.2018.02588 30619000 PMC6305618

[B14] Diener E. Fujita F. (1995). Resources, personal strivings, and subjective well-being: a nomothetic and idiographic approach. *J. Pers. Soc. Psychol.* 68 926–935. 10.1037/0022-3514.68.5.926 7776188

[B15] DiFonzo N. Bordia P. (2007). Rumor, gossip and urban legends. *Diogenes* 54 19–35. 10.1177/0392192107073433

[B16] Dunbar R. I. M. Marriott A. Duncan N. D. C. (1997). Human conversational behavior. *Hum. Nat.* 8 231–246. 10.1007/BF02912493 26196965

[B17] Ellwardt L. Labianca G. Wittek R. (2012a). Who are the objects of positive and negative gossip at work? A social network perspective on workplace gossip. *Soc. Netw.* 34 193–205. 10.1016/j.socnet.2011.11.003

[B18] Ellwardt L. Wittek R. Wielers R. (2012b). Talking about the boss: effects of generalized and interpersonal trust on workplace gossip. *Group Organ. Manag.* 37 521–549. 10.1177/1059601112450607

[B19] Farley S. D. Timme D. R. Hart J. W. (2010). On Coffee Talk and Break-Room Chatter: perceptions of Women Who Gossip in the Workplace. *J. Soc. Psychol.* 150 361–368. 10.1080/00224540903365430 20718221

[B20] Foster E. K. (2004). Research on gossip: taxonomy, methods, and future directions. *Rev. Gen. Psychol.* 8 78–99. 10.1037/1089-2680.8.2.78

[B21] Fox S. Stallworth L. E. (2005). Racial/ethnic bullying: exploring links between bullying and racism in the US workplace. *J. Vocat. Behav.* 66 438–456. 10.1016/j.jvb.2004.01.002

[B22] Gkorezis P. Petridou E. Xanthiakos P. (2014). Leader positive humor and organizational cynicism: LMX as a mediator. *Leadersh. Organ. Dev. J.* 35 305–315. 10.1108/LODJ-07-2012-0086

[B23] Greenhaus J. H. Beutell N. J. (1985). Sources of conflict between work and family roles. *Acad. Manag. Rev.* 10 76–88. 10.5465/amr.1985.4277352

[B24] Grosser T. J. Lopez-Kidwell V. Labianca G. Ellwardt L. (2012). Hearing it through the grapevine: positive and negative workplace gossip. *Organ. Dyn.* 41 52–61. 10.1016/j.orgdyn.2011.12.007

[B25] Hobfoll S. E. (1989). Conservation of resources: a new attempt at conceptualizing stress. *Am. Psychol.* 44 513–524. 10.1037/0003-066x.44.3.513 2648906

[B26] Hobfoll S. E. (2001). The influence of culture, community, and the nested-self in the stress process: advancing Conservation of Resources theory. *Appl. Psychol.* 50 337–370. 10.1111/1464-0597.00062

[B27] Hobfoll S. E. Halbesleben J. Neveu J.-P. Westman M. (2018). Conservation of resources in the organizational context: the reality of resources and their consequences. *Ann. Rev. Organ. Psychol. Organ. Behav.* 5 103–128. 10.1146/annurev-orgpsych-032117-104640

[B28] Hobfoll S. E. Lilly R. S. (1993). Resource conservation as a strategy for community psychology. *J. Comm. Psychol.* 21 128–148. 10.1002/1520-6629(199304)21:2<128::aid-jcop2290210206>3.0.co;2-5

[B29] Hofstede G. (1991). *Cultures and Organizations: Software of the Mind.* London: McGraw-Hill.

[B30] Jiang L. X. Xu X. H. Hu X. W. (2019). Can Gossip Buffer the Effect of Job Insecurity on Workplace Friendships? *Int. J Environ. Res. Public Health* 16:1285. 10.3390/ijerph16071285 30974818 PMC6479991

[B31] Keashly L. Harvey S. (2005). “Emotional abuse in the workplace,” in *Counterproductive Work Behavior: Investigations of Actors and Targets*, eds Fox S. Spector P. E. (Washington, DC: American Psychological Association), 201–235.

[B32] Kessler R. C. Andrews G. Colpe L. J. Hiripi E. Mroczek D. K. Normand S. L. T. (2002). Short screening scales to monitor population prevalences and trends in non-specific psychological distress. *Psychol. Med.* 32 959–976. 10.1017/s0033291702006074 12214795

[B33] Knecht M. Bauer G. Gutzwiller F. Hämmig O. (2011). Persistent work-life conflict and health satisfaction - A representative longitudinal study in Switzerland. *BMC Public Health* 11:271. 10.1186/1471-2458-11-271 21529345 PMC3103457

[B34] Kniffin K. M. Wilson D. S. (2005). Utilities of gossip across organizational levels - Multilevel selection, free-riders, and teams. *Hum. Nat. Interdiscipl. Biosoc. Perspect.* 16 278–292. 10.1007/s12110-005-1011-6 26189751

[B35] Kniffin K. M. Wilson D. S. (2010). Evolutionary Perspectives on Workplace Gossip: why and How Gossip Can Serve Groups. *Group Organ. Manag.* 35 150–176. 10.1177/1059601109360390

[B36] Kong M. (2018). Effect of Perceived Negative Workplace Gossip on Employees’. Behaviors. *Front. Psychol.* 9:1112. 10.3389/fpsyg.2018.01112 30050479 PMC6052122

[B37] Kossek E. E. Ozeki C. (1998). Work-family conflict, policies, and the job-life satisfaction relationship: a review and directions for organizational behavior-human resources research. *J. Appl. Psychol.* 83 139–149. 10.1037//0021-9010.83.2.139

[B38] Kuo C. C. Wu C. Y. Lin C. W. (2018). Supervisor workplace gossip and its impact on employees. *J. Manag. Psychol.* 33 93–105. 10.1108/jmp-04-2017-0159

[B39] Li H. Zou Y. Wang J. Yang X. (2016). Role of stressful life events, avoidant coping styles, and neuroticism in online game addiction among college students: a moderated mediation model. *Front. Psychol.* 7:1794. 10.3389/fpsyg.2016.01794 27920734 PMC5118950

[B40] Liu J. Kwan H. K. Lee C. Hui C. (2013). Work-to-family spillover effects of workplace ostracism: the role of work-home segmentation preferences. *Hum. Resour. Manag.* 52 75–93. 10.1002/hrm.21513

[B41] Liu X.-Y. Kwan H. K. Zhang X. (2020). Introverts maintain creativity: a resource depletion model of negative workplace gossip. *Asia Pacific J. Manag.* 37 325–344. 10.1007/s10490-018-9595-7

[B42] Lu F.-Y. Yang W.-J. Zhang Q.-L. Qiu J. (2017). Thought control ability is different from rumination in explaining the association between neuroticism and depression: a three-study replication. *Front. Psychol.* 8:838. 10.3389/fpsyg.2017.00838 28620326 PMC5450412

[B43] Matthews R. A. Wayne J. H. Ford M. T. (2014). A work–family conflict/subjective well-being process model: a test of competing theories of longitudinal effects. *J. Appl. Psychol.* 99 1173–1187. 24773400 10.1037/a0036674

[B44] Menard S. (2002). *Longitudinal Research (2nd ed.).* Thousand Oaks, CA: Sage.

[B45] Michelson G. Mouly S. (2000). Rumour and gossip in organisations: a conceptual study. *Manag. Decision* 38 339–346. 10.1108/00251740010340508

[B46] Michelson G. van Iterson A. Waddington K. (2010). Gossip in organizations: contexts. consequences, and controversies. *Group Organ. Manag.* 35 371–390. 10.1177/1059601109360389

[B47] Noon M. Delbridge R. (1993). News from Behind My Hand: gossip in Organizations. *Organ. Stud.* 14 23–36. 10.1177/017084069301400103

[B48] Porath C. L. Pearson C. M. (2010). The cost of bad behavior. *Organ. Dyn.* 39 64–71.

[B49] Ritter K.-J. Matthews R. A. Ford M. T. Henderson A. A. (2016). Understanding role stressors and job satisfaction over time using adaptation theory. *J. Appl. Psychol.* 101 1655–1669. 27537676 10.1037/apl0000152

[B50] Rosnow R. L. Esposito J. L. Gibney L. (1988). Factors influencing rumor spreading - replication and extension. *Lang. Commun.* 8 29–42. 10.1016/0271-5309(88)90004-3

[B51] Salin D. (2001). Prevalence and forms of bullying among business professionals: a comparison of two different strategies for measuring bullying. *Eur. J. Work Organ. Psychol.* 10 425–441. 10.1080/13594320143000771

[B52] Schafer R. B. (1980). Role strain and depression in two-job families. *Fam. Relat.* 29:483.

[B53] Shackelford T. K. (1997). “Perceptions of betrayal and the design of the mind,” in *Evolutionary social Psychology*, eds Kenrick D. T. Simpson J. A. (Hillsdale, NJ, US: Lawrence Erlbaum Associates, Inc), 73–108.

[B54] Shafer A. B. (1999). Brief bipolar makers for the five factor model of personality. *Psychol. Rep.* 84 1173–1179. 10.2466/pr0.84.3.1173-1179

[B55] Snyder L. A. Chen P. Y. Grubb P. L. Roberts R. K. Sauter S. L. Swanson N. G. (2005). Workplace aggression and violence against individuals and organizations: causes, consequences, and interventions. *Res. Occup. Stress Well Being* 4 1–65. 10.1016/S1479-3555(04)04001-6

[B56] Stone E. F. Hollenbeck J. R. (1989). Clarifying some controversial issues surrounding statistical procedures for detecting moderator variables - empirical-evidence and related matters. *J. Appl. Psychol.* 74 3–10. 10.1037/0021-9010.74.1.3

[B57] Tetrick L. Buffardi L. (2006). “Work-life balance: a psychological perspective,” in *Measurement Issues in Research on the Work-Home Interface*, eds Jones F. Burke R. J. Westman M. (New York, NY: Psychological Press).

[B58] Uglanova E. A. Staudinger U. M. (2013). Zooming in on life events: is hedonic adaptation sensitive to the temporal distance from the event? *Soc. Indicat. Res.* 111 265–286.

[B59] Wu L. Z. Birtch T. A. Chiang F. F. T. Zhang H. N. (2018). Perceptions of negative workplace gossip: a self-consistency theory framework. *J. Manag.* 44 1873–1898. 10.1177/0149206316632057

[B60] Wu X. Kwan H. K. Wu L. Z. Ma J. (2018). The effect of workplace negative gossip on employee proactive behavior in china: the moderating role of traditionality. *J. Bus. Ethics* 148 801–815. 10.1007/s10551-015-3006-5

[B61] Wu L. Z. Yim H. K. Kwan H. K. Zhang X. (2012). Coping with workplace ostracism: the roles of ingratiation and political skill in employee psychological distress. *J. Manag. Stud.* 49 178–199. 10.1111/j.1467-6486.2011.01017.x

[B62] Zhou A. Q. Liu Y. Su X. Xu H. Y. (2019). Gossip fiercer than a tiger: effect of workplace negative gossip on targeted employees’ innovative behavior. *Soc. Behav. Pers.* 47:e5727 10.2224/sbp.5727

